# Exploring multilocus associations of inflammation genes and colorectal cancer risk using hapConstructor

**DOI:** 10.1186/1471-2350-11-170

**Published:** 2010-12-03

**Authors:** Karen Curtin, Roger K Wolff, Jennifer S Herrick, Ryan Abo, Martha L Slattery

**Affiliations:** 1 Epidemiology, Department of Internal Medicine, University of Utah Health Sciences Center, Salt Lake City, Utah, USA; 2 Genetic Epidemiology, Department of Internal Medicine, University of Utah Health Sciences Center, Salt Lake City, Utah, USA

## Abstract

**Background:**

In candidate-gene association studies of single nucleotide polymorphisms (SNPs), multilocus analyses are frequently of high dimensionality when considering haplotypes or haplotype pairs (diplotypes) and differing modes of expression. Often, while candidate genes are selected based on their biological involvement in a given pathway, little is known about the functionality of SNPs to guide association studies. Investigators face the challenge of exploring multiple SNP models to elucidate which variants, independently or in combination, might be associated with a disease of interest. A data mining module, hapConstructor (freely-available in Genie software) performs systematic construction and association testing of multilocus genotype data in a Monte Carlo framework. Our objective was to assess its utility to guide statistical analyses of haplotypes within a candidate region (or combined genotypes across candidate genes) beyond that offered by a standard logistic regression approach.

**Methods:**

We applied the hapConstructor method to a multilocus investigation of candidate genes involved in pro-inflammatory cytokine IL6 production, *IKBKB*, *IL6*, and *NFKB1 *(16 SNPs total) hypothesized to operate together to alter colorectal cancer risk. Data come from two U.S. multicenter studies, one of colon cancer (1,556 cases and 1,956 matched controls) and one of rectal cancer (754 cases and 959 matched controls).

**Results:**

HapConstrcutor enabled us to identify important associations that were further analyzed in logistic regression models to simultaneously adjust for confounders. The most significant finding (nominal *P *= 0.0004; false discovery rate *q *= 0.037) was a combined genotype association across *IKBKB *SNP rs5029748 (1 or 2 variant alleles), *IL6 *rs1800797 (1 or 2 variant alleles), and *NFKB1 *rs4648110 (2 variant alleles) which conferred an ~80% decreased risk of colon cancer.

**Conclusions:**

Strengths of hapConstructor were: systematic identification of multiple loci within and across genes important in CRC risk; false discovery rate assessment; and efficient guidance of subsequent logistic regression analyses.

## Background

In candidate gene association studies, multiple single nucleotide polymorphisms (SNPs) selected to tag variation within a region or chosen based on previous research are usually analyzed independently, in multiSNP haplotypes, and in multiSNP genotype combinations across genes that may be functionally related. Multi-locus analyses are often of high dimensionality, especially when considering whether to analyze haplotype or diplotype data and differing modes of expression, i.e. additive, dominant, or recessive. Studying subsets of SNPs using hapConstructor, freely-available software that performs a systematic and exhaustive construction and testing of multilocus data in a Monte Carlo framework, can guide these analyses to more effectively define the haplotypes or combined genotypes on which susceptibility variants reside [1,2]. As a novel application of the hapConstructor method, we explored multilocus associations in 16 tagging-SNPs across three genes that are functionally related, I-Kappa-B Kinase-Beta (*IKBKB)*, Interleukin 6 (*IL6*), and Nuclear Factor Kappa-B, Subunit 1 (*NFKB1*) in two multicenter U.S. case-control studies of colon and rectal cancer. Within these genes, SNPs were genotyped to comprehensively tag genetic variation. Significant single SNP and multi-SNP haplotype or combined genotype associations identified in hapConstructor were carried forward to logistic regression models to simultaneously adjust for covariates. The utility of hapConstructor to guide a multilocus investigation is its integrated capability to systematically: infer phase from genotype data, model all possible haplotypes within genes, and model combined genotypes across genes (for differing inheritance modes). Thus, the potentially tedious process of setting up and evaluating all possible multilocus analyses using procedures in statistical software (such as SAS or R) can be avoided.

The inflammatory process is thought to be a key underlying component of colorectal cancer (CRC) that is initiated by pro-inflammatory cytokines such as IL-6 in response to an inflammatory insult. IL-6 stimulates secretion of C-reactive protein, an important biomarker for pro-inflammatory status in several diseases, and polymorphisms in the promoter region have been associated with C-reactive protein circulating levels. We previously reported that variants in *IL6*, dbSNP ID rs1800795 and rs1800796, were associated with reduced risk of colon cancer among recent aspirin/nonsteroidal anti-inflammatory drug (NSAID) users [3]; the rs1800795 promoter SNP, which is in linkage disequilibrium (LD) with rs1800797 (r^2 ^= 0.92), appeared to be greatest for *TP53 *mutations while rs1800796 appeared to influence all tumor types among NSAID users. Although *IKBKB *and *NFKB1 *polymorphisms have not been studied comprehensively with CRC, there is strong evidence of their contribution to the inflammation process. Nuclear transcription factor NF-κB is involved in regulation of cytokines such as IL-6 [4]. In one study, Landi, et al. evaluated a non-functional polymorphism in *NFKB1 *and did not observe a significant association with colon cancer [5].

To demonstrate its utility, we applied hapConstructor in a multilocus mining of 4 candidate intronic SNPs in *IKBKB*: rs2272733, rs3747811, rs5029748, and rs10958713; 5 SNPs in *IL6*: rs1800796, rs1800797, and rs2069827 (promoter); rs2069840 (intron), and rs2069860 (D162V); and 6 intronic SNPs in *NFKB1*: rs230510, rs3821958, rs4648090, rs4648110, rs4648127, rs11722146, and rs13117745 to comprehensively identify haplotype and composite genotype subsets of these 16 candidate inflammation-related loci associated with colon or rectal cancer. Using hapConstructor individual and multi-SNP results to focus our analyses, we further assessed these associations in multivariate logistic regression models, adjusting for potential confounders, in a large U.S. multicenter study of colon cancer (1,556 cases and 1,956 matched controls) and of rectal cancer (754 cases and 959 matched controls).

## Methods

### Study populations

Data for the study come from two case-control studies that used identical methods to recruit and interview study participants. Cases of first primary colon cancer (ICD-O 2nd edition codes 18.0, 18.2-18.9) diagnosed between 1 October, 1991 and 30 September, 1994 conducted in the Northern California Kaiser Permanent Medical Care Program (KPMCP), the Wasatch Front area of Utah, and the Twin Cities Metropolitan area of Minnesota were included in the first study. A subsequent study included incident cases diagnosed with a first primary tumor in the rectosigmoid junction or rectum identified between May 1997 and May 2001 in KPMCP and the state of Utah. Case eligibility was determined by the Surveillance Epidemiology and End Results Cancer Registries in Northern California and in Utah and the Minnesota Cancer Surveillance System (colon cancer cases only). Case eligibility included being between 30 and 79 years of age at the time of diagnosis, English speaking, mentally competent to complete the interview, no previous history of CRC, and no known (as indicated on the pathology report or previous medical history) familial adenomatous polyposis, ulcerative colitis, or Crohn's disease. Of cases contacted, 83% participated at KPMCP, 76% in Utah, and 67% in Minnesota. In the rectal cancer study, the participation rates were 75% of cases from KPCMP and 70% of cases from Utah. The studies were approved by the University of Utah Institutional Review Board as well as Institutional Review Boards at the Kaiser Permanente Medical Care Program of Northern California (KPMCP) and the University of Minnesota.

Controls, subject to the same eligibility criteria as cases, were frequency matched to cases by sex and by 5-year age cohort. At KPMCP, controls were randomly selected from membership lists. In Utah, controls 65 years and older were randomly selected from lists provided by the Centers for Medicare and Medicaid Services and controls younger than 65 were randomly selected from driver's license lists. In Minnesota, controls were randomly selected from driver's license lists. Of controls contacted for the colon cancer study, 73% participated at KPMCP, 53% participated from Minnesota and 69% participated from Utah. In the rectal cancer study, participation rates were 70% for KPMCP and 67% for Utah. The study populations have been previously described in detail [6,7]. Characteristics of colon and rectal cancer study participants are shown in Table 1.

**Table 1:** Characteristics of colon cancer and rectal cancer studies

	**Colon study**				**Rectal study**			
		
	**Cases**		**Controls**		**Cases**		**Controls**	
				
**Characteristic**	**n**	**%**	**n**	**%**	**n**	**%**	**n**	**%**
			
Total	1556		1956		754		959	
								
Male	871	56.0	1047	53.5	451	59.8	541	56.4
Female	685	44.0	909	46.5	303	40.2	418	43.6
								
Age at diagnosis or selection								
30-39	23	1.5	40	2.0	19	2.5	21	2.2
40-49	102	6.6	128	6.5	96	12.7	101	10.5
50-59	290	18.6	326	16.7	196	26.0	243	25.3
60-69	538	34.6	673	34.4	250	33.2	329	34.3
70-79	603	38.8	789	40.3	193	25.6	265	27.6
								
Center								
No. California (KPMCP)	744	47.8	787	40.2	480	63.7	594	61.9
Utah	250	16.1	378	19.3	274	36.3	365	38.1
Minnesota	562	36.1	791	40.4	0	-	0	-
								
White, non-Hispanic	1429	91.8	1828	93.5	625	82.9	824	85.9
White, Hispanic	59	3.8	75	3.8	61	8.1	63	6.6
Black	68	4.4	53	2.7	29	3.8	43	4.5
Asian	0	-	0	-	39	5.2	29	3.0

### Data collection

Trained and certified interviewers collected diet and lifestyle data [8]. The referent year for the study was the calendar year approximately 2 years prior to date of diagnosis (cases) or selection (controls). Information was collected on demographic factors such as age, sex, and study center; diet, physical activity, aspirin and non-steroidal drug use, body size, and other lifestyle factors including medical, family, and reproductive history. Participants were asked to report their race/ethnicity; most reported they were white, non-Hispanic (Table 1). The data collection methods of these studies have been detailed previously [6,7].

### Genotyping

All markers were genotyped using a multiplexed bead array assay format based on GoldenGate chemistry (Illumina, San Diego, California). A genotyping call rate of 99.85% was attained. Two-hundred-fifty-three internal replicates representing 4.4% of the sample set were blinded and included. The duplicate sample genotype concordance rate was 100.00% as determined by 350,070 matching genotypes among sample pairs.

Polymorphisms on the custom Illumina platform were selected to account for genetic variation within each candidate gene with redundant SNPs genotyped in most tagging-SNP linkage disequilibrium bins. Of 7 markers in *IKBKB*, one SNP failed genotyping, one SNP was monomorphic in our study populations, and one SNP failed Hardy-Weinberg equilibrium (HWE) testing resulting in 4 SNPs carried forward to analysis. Of 6 markers in *IL6*, one SNP (rs1800795) was in high LD with another promoter SNP (rs1800797) and was not carried forward to analysis, resulting in 5 *IL6 *SNPs. Of 18 *NFKB1 *markers, one SNP failed genotyping, one very rare SNP had a MAF < 0.01, and 9 SNPs were redundant and well-represented by another binned tagging-SNP with fewer missing values in the data (pairwise r^2 ^> 0.80); thus 7 SNPs were carried forward to analysis, for a total of 16 SNPs. A description of genotyped markers (in chromosome order) in *IKBKB*, *IL6*, and *NFKB1 *(including MAF and *P*-HWE) is shown in Additional file 1. A total of 5,225 samples were successfully genotyped: 1,956 controls and 1,556 cases, colon study; 959 controls and 754 cases, rectal study.

### Statistical methods

The hapConstructor 1.0 module of Genie 2.7.2.1, a freely available software package [2], was used to comprehensively analyze multi-locus SNP haplotypes and combined genotypes to identify associations with affected status and assess their significance in a Monte Carlo framework, as previously detailed [1]. Briefly, all single SNP associations are tested independently. In each subsequent forward step, a SNP is added to two-locus through n-locus SNP sets whose test of association of an empirical *P*-value exceeds a user-defined threshold at the previous step. Rather than specify a significance threshold that remained constant at each step, we decided a priori to use successively more stringent empirical *P*-value significance thresholds (10,000 simulations), as the number of SNPs within each multilocus subset was increased: 0.05, 0.01, 0.001 for single, two, and three-locus sets, respectively; and 0.0001 for ≥ 4 locus sets. First, all single SNPs are tested for association with colon and rectal cancer. For any SNP that reaches a single SNP *P*-value threshold of 0.05, the second step considers all 2-locus SNP sets containing the significant SNP. Any 2-locus haplotypes or composite genotypes that then surpass a 2-locus threshold of 0.01 are carried forward to a third step which considers all 3-locus SNP sets that include the two markers. Any 3-locus subsets that surpass a threshold of 0.001 are carried forward to an analysis of 4-locus subsets, and so forth until there are no subsets that satisfy a subsequent threshold of 0.0001. This feature allows the user to alter the significance level at each subsequent step, analogous to data mining procedures used in SNP association studies such as hill climbing that allow the "best" models to move forward or a greedy algorithm that performs an optimization [9,10]. More stringent *P *values with each progressive step also assured that multi-locus models were adding statistically beyond significance observed for a single SNP or combinations of SNPs from a previous step, and multilocus models that achieve significance are focused on those more likely to identify a significant haplotype association or interaction across genes after accounting for multiple comparisons. Thus, in our genetic association investigation and in those of others [11-13], we find increasing the stringency of the significance level as the model becomes more complex is appropriate in moving toward a more tractable solution given the large number of comparisons.

For each gene, the full-length haplotype frequencies were estimated from genotype data based on an expectation-maximization algorithm [14], and the maximum likelihood estimate haplotype pairs for each subject were used for analysis. For two-locus haplotype combinations from biallelic data, each set of 4 possible two-locus haplotypes (assuming linkage equilibrium) is modeled as additive, dominant, or recessive (12 haplotype tests). To minimize inferred haplotypes from missing genotypes, samples missing data in *IKBKB*, *IL6*, or *NFKB1 *were excluded from within-gene analyses and samples missing any data for these 3 genes were excluded from analyses across all markers. To avoid sparse cell sizes, hapConstructor examines composite genotypes modeled as dominant and recessive, but not additive. Composite genotype models include all dominant and recessive combinations across loci; assuming linkage equilibrium, 4 composite genotypes can be tested across any two SNPs: dominant/dominant, dominant/recessive, recessive/dominant, or recessive/recessive. If the underlying mode of inheritance is additive, a dominant model performs adequately. Summaries of all tests were stored, and a construction-wide false discovery rate (FDR) that accounts for the multi-SNP process was generated, as described [1]. All *P*-values for individual odds ratio test statistics in hapConstructor were empirically derived based on 10,000 simulations in the Genie null distribution as detailed previously [15]. Although the study population was predominantly white and non-Hispanic, separate genotype files by race/ethnicity to account for allele frequency differences were incorporated using the meta-analysis capabilities of Genie based on Cochran-Mantel-Haenszel techniques as previously detailed [16].

SAS^® ^statistical package, version 9.2 (Cary, N.C.) was used to conduct logistic regression analyses to simultaneously consider covariates. Logistic regression models were used to estimate odds ratios (OR) and 95% confidence intervals (CI) for associations between independent genotypes and multilocus SNP sets identified as significantly associated in hapConstructor in colon or rectal cancer. Logistic regression models were adjusted for variables used to match controls to cases: age at diagnosis or selection, state of residence (center), sex, and race [17]. Additionally, logistic regression models were adjusted for potential confounders: smoking, use of nonsteriodal anti-inflammatory medications, body mass index, and family history; however, these variables did not substantively impact estimates, and thus were not included. For gene-gene interaction logistic regression models (Additional file 2), *P *for interaction was determined by comparing a full model including an ordinal multiplicative interaction term to a reduced model without an interaction term, using a likelihood ratio test [18].

## Results

Our findings from hapConstructor and logistic regression analyses are presented as follows: first, associations of single SNPs analyzed independently; second, associations of multilocus haplotypes within each gene; third, associations of multilocus composite genotypes across genes including an illustration of the hapConstructor method and its use in addressing multiple comparisons.

### Single SNP results

In the hapConstructor analysis used to systematically explore associations for haplotypes within *IKBKB*, *IL6*, and *NFKB1 *and composite SNP genotypes across genes, we generally observed similar associations for the same single-locus SNPs significant at the 0.05 level in hapConstructor as for a logistic regression analysis of each SNP considered independently (Table 2). In colon cancer, independent variant alleles in all three genes appear to decrease risk of colon cancer. Conversely, in rectal cancer, only *NFKB1 *SNPs were independently associated, with 3 of 4 polymorphisms increasing risk. Additional file 1 includes a complete listing of ORs and 95%CIs from logistic regression models for all independent SNPs in *IKBKB*, *IL6*, and *NFKB1 *and risk of colon and rectal cancer, minimally adjusted for age, sex, race and center.

**Table 2:** Single SNP results from hapConstructor and from multivariable-adjusted logistic regression

			Genetic	hapConstructor*		Logistic regression**	
Gene	SNP	Comparison	Model	OR	*P*	OR	95%CI
**Colon study**							
*IL6*	rs2069860	AT/TT vs. AA	Dominant	0.53	0.02	0.55	0.32, 0.95
*IKBKB*	rs2272733	TT vs. CC	Additive	0.55	0.02	0.56	0.34, 0.93
*NFKB1*	rs4648110	AA vs. TT	Additive	0.63	0.01	0.65	0.44, 0.94
		AA vs. TT/TA	Recessive	0.64	0.01	0.66	0.45, 0.96
	rs13117745	TT vs. CC	Additive	0.61	0.03	0.61	0.37, 1.00
		TT vs. CC/CT	Recessive	0.62	0.04	0.64	0.39, 1.04
**Rectal study**							
*NFKB1*	rs230510	TT vs. AA	Additive	0.66	0.004	0.65	0.49, 0.87
		TT vs. AA/AT	Dominant	0.77	0.009	0.79	0.51, 0.94
	rs3821958	GG vs. AA	Additive	1.34	0.04	1.32	1.00, 1.75
	rs11722146	GA/AA vs. GG	Dominant	1.21	0.047	1.24	1.03, 1.51
	rs13117745	TT vs. CC	Additive	1.92	0.04	1.69	0.93, 3.07

NOTES: Odds ratios (ORs) compared to reference of major allele homozygotes; 95% confidence interval (95% CI)

*adjusted for race

**adjusted for age, sex, center, and race

### Haplotype results

Any SNP identified as significantly associated with colon and rectal cancer in the first step was then analyzed in 2-locus haplotypes with all other markers within *IKBKB, IL6*, and *NFKB1*. We did not observe any informative haplotypes within *IKBKB *or *IL6 *using hapConstructor that were associated with colon cancer beyond the single SNP results, nor in rectal cancer. Using hapConstructor, we were able to identify significant 2-locus *NFKB1 *haplotypes carried forward to subsequent logistic regression analyses of cancer risk as shown in Table 3 by study, in order of significance. Although hapConstructor is a data mining tool that can systematically assess multilocus associations within candidate genes or regions, a determination by the researcher of the informativeness of any haplotype of interest is nonetheless warranted. In relation to *NFKB1 *and risk of colon cancer, the most significant single locus identified in hapConstructor was rs4648110 (Table 2). In significant two-locus models, a second SNP, either rs230510 (A) or rs13117745 (T), was carried in a haplotype with the rs4648110 minor allele (Table 3). However, haplotype ORs did not differ substantively from the effect size of carrying variant rs4648110 or rs13117745 alleles, when the SNP genotypes were considered alone (*P *= 0.009; FDR *q *= 0.187). Pairwise correlation between this marker and rs13117745 was r^2 ^= 0.71, and thus may be the same association result with an underlying disease locus. Pairwise correlation between rs4648110 and rs230510 was low (r^2 ^= 0.18), however carriage of the rs230510 major allele in a haplotype with the rs4648110 variant likewise did not substantively alter risk. Similarly in rectal cancer, the most significantly associated *NFKB1 *SNP, rs230510, appears to independently contribute to decreased risk with no additional information in terms of either effect size or significance, conferred by a second locus (Tables 2 and 3). The most nominally significant 2-locus haplotype associated with rectal cancer (*P *= 0.0028; FDR *q *= 0.074) was across rs11722146 and rs4648110 (r^2 ^= 0.10) in which carrying two copies of the minor alleles A-A confers a ~50% increased risk, which is somewhat greater in effect than an OR of 1.2 when rs11722146 A allele was considered independently. There were no haplotypes beyond the 2-locus step that were significant at more stringent thresholds.

**Table 3:** NFKB1 haplotypes and associations with colon and rectal cancer from hapConstructor and from multivariable-adjusted logistic regression

			Genetic	hapConstructor*		Logistic regression**	
SNP 1	SNP 2	Haplotype	Model	OR	*P*	OR	95%CI
**Colon study**							
rs230510 A > T	rs4648110 T > A	2 copies A-A vs. 0/1 copy	Rec.	0.62	0.009	0.65	0.45, 0.95
rs13117745 C > T	rs4648110 T > A	2 copies T-A vs. 0 copies	Additive	0.62	0.009	0.65	0.44, 0.94
**Rectal study**							
rs3821958 A > G	rs230510 A > T	2 copies A-T vs. 0 copies	Additive	0.66	0.006	0.68	0.50, 0.91
rs4648127 C > T	rs230510 A > T	2 copies C-T vs. 0 copies	Additive	0.66	0.006	0.68	0.50, 0.91
rs11722146 G > A	rs230510 A > T	2 copies G-T vs. 0 copies	Additive	0.66	0.006	0.67	0.50, 0.90
rs13117745 C > T	rs230510 A > T	2 copies C-T vs. 0 copies	Additive	0.66	0.006	0.67	0.50, 0.90
rs230510 A > T	rs4648090 G > A	2 copies T-G vs. 0 copies	Additive	0.67	0.007	0.68	0.51, 0.90
rs11722146 G > A	rs4648090 G > A	2 copies A-A vs. 0 copies	Additive	1.47	0.007	1.47	1.11, 1.93
rs230510 A > T	rs4648110 T > A	2 copies T-T vs. 0 copies	Additive	0.68	0.009	0.68	0.51, 0.91
rs3821958 A > G	rs4648110 T > A	2 copies G-A vs. 0 copies	Additive	1.52	0.005	1.49	1.12, 1.99
rs11722146 G > A	rs13117745 C > T	2 copies A-T vs. 0 copies	Additive	1.51	0.005	1.53	1.16, 2.01
rs11722146 G > A	rs4648127 C > T	2 copies A-T vs. 0 copies	Additive	1.33	0.004	1.36	1.12, 1.65
rs3821958 A > G	rs13117745 C > T	2 copies G-T vs. 0 copies	Additive	1.53	0.003	1.52	1.13, 2.02
rs11722146 G > A	rs4648110 T > A	2 copies A-A vs. 0 copies	Additive	1.54	0.0028	1.55	1.17, 2.04

NOTES: Odds ratio (OR); 95% confidence interval (95% CI); Recessive (Rec)

*adjusted for race

**adjusted for age, sex, center, and race; all *P*-trends (additive model) were significant, <0.01

### Composite genotype results

In complex disease pathways in which it is hypothesized a number of genes of modest effect work together to influence risk, composite genotypes across markers residing on different chromosomes may be important. Similar to hapConstructor steps outlined above for investigating SNP haplotypes within genes, markers across all three genes were analyzed jointly. Any significant 2-SNP composite genotypes in hapConstructor are brought forward into an analysis of 3 SNPs etc., using successively more stringent significance thresholds in each step. We were able to identify genotype combinations that appear to influence risk of colon and rectal cancer using hapConstructor for SNPs across *IKBKB*, *IL6*, and *NFKB1*. In colon cancer, a two-SNP composite genotype comprised of *IL6 *rs1800797 (dominant model) and *NFKB1 *rs4648110 (recessive model) was significantly associated with 60% decreased risk (*P *= 0.0004; FDR *q *= 0.037). At the three-locus construction step, a combined genotype also including *NFKB1 *rs13117745 was of the same magnitude in effect, with a somewhat greater nominal significance (*P *= 0.0002). The presence of LD between the two *NFKB1 *markers (rs1311745 and rs4648110) in this three-locus set was previously noted. The strongest hapConstructor association (also significant after accounting for multiple tests) was observed across all 3 inflammation-related genes, *IKBKB *(rs5029748) *IL6 *(rs1800797), and *NFKB1 *(rs4648110). Individuals with 1 or 2 minor alleles at rs5029748 and rs1800797, combined with 2 minor alleles at rs4648110, had an almost 80% decreased colon cancer risk (*P *= 0.0004; FDR *q *= 0.037). Although this composite genotype profile was relatively rare in our population (~1% of subjects), carriers of major alleles at these common loci appear to be at greater risk of disease. There were no combinations of ≥ 4 SNPs in colon study participants that met hapConstructor significance thresholds.

In rectal cancer, only two-locus composite genotypes across *IKBKB *and *NFKB1*, and *IL6 *and *NFKB1 *markers met significance thresholds. The most significant findings were *IL6 *rs1800797 (dominant model) and either *NFKB1 *rs11722146 or rs3821958 (also modeled as dominant). However, adjusting for multiple tests the observed significance may represent a chance finding (FDR *q *= 0.44). In terms of effect size, the odds ratios do not differ substantively from single SNP results although in logistic regression models adjusted for covariates, *IKBKB *rs3747811 and *IL6 *rs1800797 may mitigate increased risk of *NFKB1*. Informative composite genotype models identified in hapConstructor were subsequently analyzed in multivariate-adjusted logistic regression models as highlighted in Table 4. Results of logistic regression models including a multiplicative interaction term for composite genotype associations identified in hapConstructor (Table 4) are shown in Additional file 2 to illustrate the utility of hapConstructor to prescreen significant SNP combinations that inform more complicated analyses.

**Table 4:** Composite genotype associations with colon and rectal cancer from hapConstructor and from multivariable-adjusted logistic regression

				Genetic	hapConstructor*		Logistic regression**	
SNP 1	SNP 2	SNP 3	SNP1/SNP2/SNP3 genotype	Model	OR	*P*	OR	95%CI
**Colon study**								
*IKBKB *rs3747811	*NFKB1 *rs4648110		(TA or AA)/AA	Dom./Rec.	0.56	0.0094	0.54	0.34, 0.87
*IL6 *rs2069827	*NFKB1 *rs4648110		(GT or TT)/AA	Dom./Rec.	0.23	0.0058	0.21	0.06, 0.74
*IL6 *rs1800797	*NFKB1 *rs4648110		(GA or AA)/AA	Dom./Rec.	0.41	0.0004	0.40	0.23, 0.72
*IKBKB *rs5029748	*IL6 *rs1800797	*NFKB1 *rs4648110	(CA or AA)/(GA or AA)/AA	Dom./Dom./Rec.	0.21	0.0004	0.20	0.06, 0.67
*IL6 *rs1800797	*NFKB1 *rs4648090	*NFKB1 *rs4648110	(GA or AA)/(GA or AA)/AA	Dom./Dom./Rec.	0.40	0.0004	0.40	0.21, 0.73
*IL6 *rs1800797	*NFKB1 *rs13117745	*NFKB1 *rs4648110	(GA or AA)/(GA or AA)/AA	Dom./Dom./Rec.	0.39	0.0002	0.39	0.21, 0.73
**Rectal study**								
*IKBKB *rs3747811	*NFKB1 *rs11722146		(TA or AA)/(GA or AA)	Dom./Dom.	1.30	0.0096	1.11	0.83, 1.50
*IL6 *rs1800797	*NFKB1 *rs11722146		(GA or AA)/(GA or AA)	Dom./Dom.	1.35	0.0068	1.38	1.04, 1.83
*IL6 *rs1800797	*NFKB1 *rs3821958		(GA or AA)/(AG or GG)	Dom./Dom.	1.34	0.0044	1.15	0.85, 1.56

NOTES: Odds ratio (OR); 95% confidence interval (95%CI); Dominant (Dom); Recessive (Rec)

*adjusted for race; reference category is all other composite genotypes

**adjusted for age, sex, center, and race; reference category is major allele homozygote (dominant) or heterozygote (recessive) for each SNP

**Figure 1:**
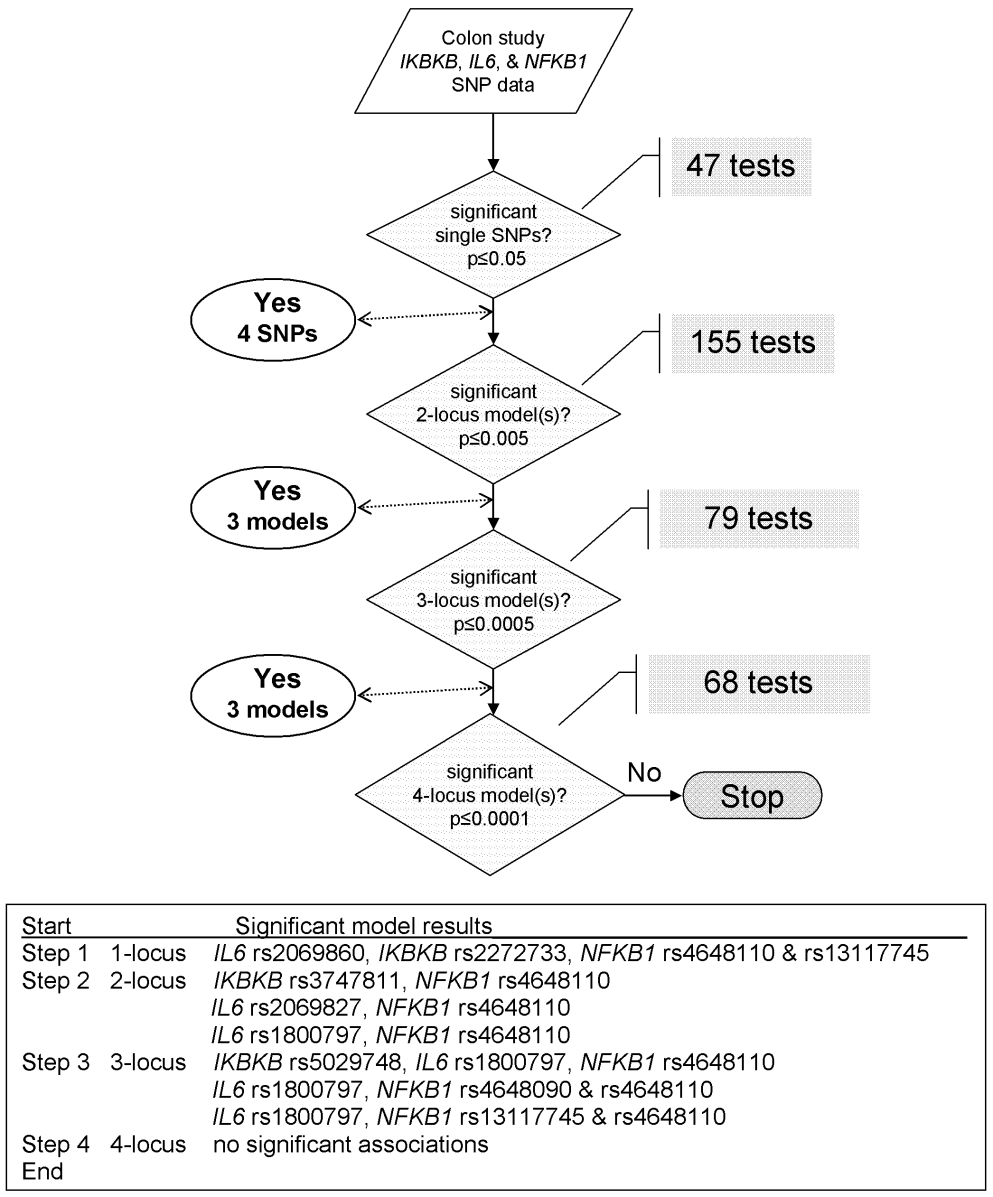
**Illustration of the hapConstructor method and associations across *IKBKB*, *IL6*, and *NFKB1 *SNPs with risk of colon cancer**.

The hapConstructor method, applied to our analysis of colon risk across genes, is illustrated in Figure 1. In the first step, 4 single SNP associations were identified that met a significance of 0.05, using a dominant, recessive and additive genotype model for each of 16 SNPs for a total of 47 tests (rs2069860 was not tested using a recessive model because there were no rare-allele homozygotes). These 4 SNPs were carried forward to step 2 and tested in 2-locus composite genotype models with each other (6 comparisons) and all 12 SNPs remaining (48 comparisons). Depending on allele frequencies and the degree of LD between SNPs, not all 4 composite-model combinations between two loci (dominant/dominant, dominant/recessive, recessive/dominant, or recessive/recessive) may have data. In our colon cancer analysis, a total of 155 tests were performed at the 2-locus step out of a possible 216 tests. There were 3 models of 4 SNPs, in 2-locus combinations, significant at the 0.01 level that were carried forward to step 3. At the 3-locus step, 79 tests were performed out of a possible 312 tests (39 comparisons and a maximum of 8 composite genotype models, various combinations of dominant or recessive at each of 3 SNPs). There were 3 models of 3-locus combinations, involving 5 SNPs, which met a significance threshold of 0.001 and thus were carried forward. At the 4-locus step, 68 tests were performed out of 576 possible tests (36 comparisons and a maximum of 16 composite genotype models). There were no 4-locus results significant below 0.0001, and thus hapConstructor did not continue further. A total of 349 tests were performed in the multilocus analyses of colon cancer across all three genes in hapConstructor as outlined, which resulted in 4 single-SNP and 6 multiple-SNP genotype models carried forward to a multivariable logistic regression. Absent a freely available and easy to implement systematic tool such as hapConstructor to guide our analysis of this pathway arm, operator effort to similarly step through an exhaustive analysis of possible combinations of loci under different modes of inheritance would be required (Table 5).

**Table 5:** Comparison of hapConstructor and logistic regression characteristics in multilocus modeling

Characteristic	hapConstructor software	Standard logistic regression procedure	Stepwise logistic regression procedure
Integrated phase estimation and building of haplotypes from genotype data	Yes	No; specialized software required	No; specialized software required

Systematically model all 2 to n-locus haplotypes within a gene	Yes	Yes, with additional programming	Yes, with additional programming

Systematically model mode of inheritance (dominant, recessive, additive)	Yes	Yes, with additional programming	Yes, with additional programming

Systematically model all SNP combinations across genes	Yes	No	No

User-specified significance levels	Yes; can vary for single locus to n loci	Yes	Yes

Model simultaneous covariates	No	Yes	Yes

Multiple testing correction	Yes (FDR)	Yes (add-on procedure)	Yes (add-on procedure)

Runtime (excluding additional operator programming time)	Slow to adequate	Fast	Fast

Each multistep hapConstructor colon or rectal association haplotype analysis, using marker files stratified by race/ethnicity, required ~30 minutes overall runtime using Java 1.6 on a Dell Precision T5500 computer with 4 gigabytes of memory, using a Windows Vista Business 64-bit operating system. A composite genotype analysis incorporating all 16 SNPs required ~90 minutes. Construction-wide *P*-value assessment and determination of an empirical FDR was considerably more time intensive due to generation of 1,000 simulated datasets (each with 10,000 null simulations) which required an additional 15-20 hours to complete for a within-gene haplotype analysis and 3-4 days to complete for the composite genotype analysis across all SNPs.

## Discussion

Proteins encoded by the genes selected for the analysis were hypothesized as working together to influence colorectal cancer risk through an inflammation-related pathway [19]. Binding of pro-inflammatory cytokines, such as IL-6, to their receptors activate the NF-κB complex which in turn activates expression of cytokines and cycloxygenase-2. Thus, NF-κB is an important nuclear transcription factor that regulates a large number of cytokines and is critical for the regulation of inflammation; increased transcription of NF-κB can increase inflammation and angiogenesis as well as cell survival and growth [20]. I-kappa-B kinase (IKK), which phosphorylates I-kappa-B proteins, is a key regulator of transcriptional activity of the NF-κB complex [21]; IκB proteins are inhibitors of NF-κB [20]. While the importance of the genes in the inflammation pathway has been well documented, our study is the first to comprehensively examine polymorphisms in either *NFKB1 *or *IKBKB *to identify functional polymorphisms associated with colorectal cancer.

For independent SNP associations, we observed associations with *IL6 *and *IKBKB *in colon cancer but not rectal cancer. *NFKB1 *SNPs appeared to be important in both cancers, however the direction of risk differed with variant alleles decreasing risk of colon cancer and, other than rs230510, increasing risk of rectal cancer (Table 2). We have previously reported associations in other genetic polymorphisms, potentially modified by lifestyle and environmental factors, that appear to influence colon and rectal cancer differentially [22-24], and thus these results are not surprising. In our hapConstructor analysis, 2-locus haplotypes within the *NFKB1 *gene were significant beyond single SNP results (Table 3); however, after considering LD between markers and adjusting for multiple comparisons, it is unlikely that a haplotype represents an independent finding beyond associations observed for single *NFKB1 *SNPs. In regard to composite genotype associations across all 3 hypothesized inflammation-related genes (Table 4), of interest is our observation that promoter variant *IL6 *rs1800797, while not independently associated, appears to be working in concert with *IKBKB *and *NFKB1 *to influence risk of colon and cancer, even after multiple testing is considered. This SNP is in high LD with a SNP in the *IL6 *regulatory region (-572 G > C; rs1800795) that was shown to functionally influence IL-6 expression and increase CRP levels [25]; a potential glucocorticoid receptor element exists nearby. As little is known about the biological relevance of SNPs in *IKBKB *and *NFKB1*, our findings will be taken forward to functionality testing. In logistic regression analyses of composite genotypes, multiplicative *P*-interaction terms for SNPs across the pathway were significant or borderline significant at the 0.05 level in most models (Additional file 2), which supports the hapConstructor finding of a joint effect.

The hapConstructor tool has been applied to the analysis of haplotypes in recent candidate gene association studies [12,26,27]. In addition to haplotypes, an attractive feature of hapConstructor is the ability to comprehensively and systematically evaluate composite genotypes of loci across multiple genes (Table 5). This feature is potentially valuable for investigations of multiple genes working together in candidate disease pathways, and our findings represent a novel application of hapConstructor in this regard; furthermore, it is not readily available in standard logistic regression procedures (Table 5). As significant SNP/SNP combinations are carried forward in successive 1 to n-locus steps and tested against remaining SNPs, we were able to efficiently capture an important composite gene association including an *IL6 *promoter SNP (rs1800797) that was not independently associated with colon or rectal cancer and may have otherwise been missed in a traditional logistic regression framework. As participants in our studies differed in race/ethnicity, we were able to utilize the meta-analysis capabilities in Genie software to appropriately assign different allele frequencies to the Monte Carlo simulation of a null distribution [16]. Although our study participants were independent population-based cases and controls, another strength is the ability of hapConstructor in the Genie package to incorporate data of related individuals in families or extended pedigrees instead of, or in addition to, unrelated cases and controls while providing valid significance testing [15,16]. Another potential strength is the ability to infer missing genotypes in hapConstructor, as the user can specify the amount of missing data allowed for each sample in haplotype estimation. However, as our analysis also focused on multiSNP associations across genes, inferring missing data from SNPs on other chromosomes was not appropriate and thus samples with missing genotypes were excluded.

A potential limitation of hapConstructor and Monte Carlo testing in general is computational burden which depends on number of SNPs under consideration, number of simulations (particularly in construction-wide assessment of significance and generation of FDR), sample size, and threshold values for significance. In our investigation of 16 loci using 10,000 simulations to provide empirical p-values in 5,225 total cases and controls, hapConstructor runtimes were acceptable, but in other situations may be considerable, depending on the number of genes and SNPs within genes. It is difficult to quantify additional operator time required to set up all possible multilocus haplotype models (within genes) and combined SNP genotype models (across genes) for various modes of inheritance. Although not pre-integrated into standard statistical packages, we acknowledge these capabilities exist but require more programming time for setup and analysis.

A multilocus mining method, hapConstructor is dependent on user-specified significance thresholds in successive 1 to n-locus steps. We chose to specify successively more stringent significance thresholds beginning with a nominal significance of 0.05 for single-locus associations, 0.01 for two-locus associations, 0.001 for three-locus associations, and 0.0001 for 4 or more loci (minimum resolution given 10,000 simulations) to focus on multilocus models that add to statistical significance beyond single SNP associations or SNP combinations identified in a previous step. Using a constant 0.05 threshold, we identified several multilocus associations that met the threshold simply because a single SNP was significant. By using successively more stringent significance levels, we can target models for further investigation that are more likely to identify a true interaction. Although the hapConstructor method can provide an odds ratio statistic and a *P *for significance, it does not simultaneously control for potential confounding variables. We nonetheless found it to be a useful tool to identify multilocus relationships for further analysis in a multivariate logistic regression framework. We believe the integrated capabilities of hapConstructor are most useful when combined with subsequent logistic regression to refine risk estimates and corresponding confidence intervals with adjustment for confounding variables, and to further test for interaction among significant multilocus associations identified in hapConstructor.

The hapConstructor method represents a data mining approach to directing a multiSNP investigation when several genes and SNPs are involved, as in our application. Other methods that reduce the dimensionality of multiSNP analyses have been recently proposed. For example, within a candidate region or gene of interest, Yang, et al. describe a haplotype-based stepwise procedure to eliminate extraneous SNPs in haplotype association analysis [28] and others have proposed principal components regression where multiple SNPs from the candidate region tend to be correlated [29,30]. Other methods have been proposed to test for potential gene-gene interactions to identify specific locus combinations of interest for further investigation, including extended multifactor dimensionality reduction [31] or computationally-efficient algorithms for filtering these interactions [32].

## Conclusions

In conclusion, hapConstructor provided a useful tool to systematically and comprehensively guide our exploration of multilocus SNP combinations in three candidate inflammation-related genes, *IKBKB*, *IL6*, and *NFKB1 *in a U.S. case-control study of colon and rectal cancer. Our findings suggest that polymorphisms in *IKBKB, IL6*, and *NFKB1 *may jointly interact to influence colon and rectal cancer risk through an inflammation-related pathway. The utility of hapConstructor was further enhanced by the capability of false discovery rate assessment to account for multiple comparisons.

## Competing interests

The authors declare that they have no competing interests.

## Authors' contributions

KC carried out the statistical analyses and drafted the manuscript. RKW directed the genotyping, and helped to draft the manuscript. JSH provided data management and statistical programming efforts, and helped to draft the manuscript. RA provided technical assistance with software and helped to draft the manuscript. MLS conceived the study, directed its design, coordination, and data collection efforts, and helped to draft the manuscript. All authors read and approved the final manuscript.

## Pre-publication history

The pre-publication history for this paper can be accessed here:

http://www.biomedcentral.com/1471-2350/11/170/prepub

## Supplementary Material

Additional file 1 **Description of candidate SNPs and association with colon or rectal cancer**. All genotyped SNPs (in chromosome order) in *IKBKB*, *IL6*, and *NFKB1 *including their minor allele frequencies and individual odds ratios (95%CIs) from logistic regression models for risk of colon and rectal cancer. Click here for file

Additional file 2 **Composite genotype logistic regression model associations with colon and rectal cancer identified in hapConstructor**. Results of logistic regression models including a multiplicative interaction term for composite genotype associations identified in hapConstructor. Click here for file
