# Supplementary material for: Exploring multilocus associations of inflammation genes and colorectal cancer risk using hapConstructor
Source: BMC Med Genet. 2010 Dec 3;11:170. doi: 10.1186/1471-2350-11-170 (PMC3006374; doi:10.1186/1471-2350-11-170)
Supplement: Additional file 1 — Description of candidate SNPs and association with colon or rectal cancer. All genotyped SNPs (in chromosome order) in IKBKB, IL6, and NFKB1 including their minor allele frequencies and individual odds ratios (95%CIs) from logistic regression models for risk of colon and rectal cancer. [file 1471-2350-11-170-S1.DOC]

Additional file 1. Description of candidate SNPs and association with colon or rectal cancer

| Gene | Chr. | dbSNP ID | Region | MAF in controls | Major/Minor Allele | *P* HWE | Colon study | Rectal study |
| --- | --- | --- | --- | --- | --- | --- | --- | --- |
|  |  |  |  |  |  |  | Minor allele homozygotes OR (95%CI) | Minor allele homozygotes OR (95%CI) |
| *IKBKB* | 8p11.2 | **rs3747811** | **intronic** | **0.48** | **T/A** | **0.97** | **1.02 (0.85, 1.24)** | **0.88 (0.67, 1.16)** |
|  |  | **rs5029748** | **intronic** | **0.27** | **C/A** | **0.60** | **1.12 (0.86, 1.46)** | **0.91 (0.63, 1.31)** |
|  |  | **rs2272733** | **intronic** | **0.13** | **C/T** | **0.95** | **0.56 (0.34, 0.93)** | **1.30 (0.68, 2.49)** |
|  |  | rs17875749 | coding |  | FAILED |  | N/A | N/A |
|  |  | **rs10958713** | **intronic** | **0.36** | **C/T** | **0.83** | **1.05 (0.85, 1.29)** | **0.94 (0.69, 1.27)** |
|  |  | rs13278372 | intronic | 0.13 | C/A | <0.01 | 0.62 (0.37, 1.02) | 1.37 (0.72, 2.61) |
|  |  | rs17611716 | coding | 1.00 | G/ -- |  | N/A | N/A |
| *IL6* | 7p21 | **rs2069827** | **UTR-5** | **0.09** | **G/T** | **0.93** | **0.86 (0.72, 1.04)*** | **0.95 (0.73, 1.25)*** |
|  |  | **rs1800797** | **UTR-5** | **0.38** | **G/A** | **0.05** | **0.93 (0.76, 1.14)** | **1.03 (0.76, 1.41)** |
|  |  | **rs1800796** | **UTR-5** | **0.07** | **G/C** | **0.69** | **0.89 (0.73, 1.10)*** | **1.26 (0.94, 1.69)*** |
|  |  | *rs1800795* | *UTR-5* | *0.39* | *G/C* | *0.08* | *0.85 (0.70, 1.04)* | *1.04 (0.76, 1.41)* |
|  |  | **rs2069840** | **intronic** | **0.35** | **C/G** | **0.89** | **0.90 (0.72, 1.12)** | **0.94 (0.69, 1.29)** |
|  |  | **rs2069860** | **coding** | **0.01** | **A/T** | **0.84** | **0.55 (0.32, 0.95)*** | **1.02 (0.52, 2.04)*** |
| *NFKB1* | 4q24 | *rs3774932* | *intronic* | *0.45* | *G/A* | *0.53* | *1.02 (0.84, 1.23)* | *0.60 (0.45, 0.80)* |
|  |  | *rs3774938* | *intronic* | *0.39* | *A/G* | *0.68* | *1.03 (0.84, 1.25)* | *1.44 (1.08, 1.91)* |
|  |  | rs230516 | intronic |  | FAILED |  | N/A | N/A |
|  |  | **rs230510** | **intronic** | **0.44** | **A/T** | **0.62** | **1.00 (0.82, 1.21)** | **0.65 (0.49, 0.87)** |
|  |  | **rs13117745** | **intronic** | **0.15** | **C/T** | **0.99** | **0.61 (0.37, 1.00)** | **1.69 (0.93, 3.07)** |
|  |  | *rs1801* | *intronic* | *0.36* | *G/C* | *0.62* | *1.05 (0.85, 1.29)* | *1.25 (0.93, 1.69)* |
|  |  | **rs3821958** | **intronic** | **0.41** | **A/G** | **0.87** | **1.03 (0.84, 1.25)** | **1.32 (1.00, 1.75)** |
|  |  | *rs3774959* | *intronic* | *0.36* | *G/A* | *0.62* | *1.05 (0.85, 1.29)* | *1.25 (0.93, 1.69)* |
|  |  | *rs4648068* | *intronic* | *0.32* | *A/G* | *0.61* | *1.08 (0.87, 1.36)* | *1.27 (0.93, 1.75)* |
|  |  | rs4648072 | coding | <0.01 | A/G | 0.85 | 0.97 (0.61, 1.54)* | 1.50 (0.73, 3.08)* |
|  |  | rs3774964 | intronic | 0.37 | A/G | 0.77 | 1.06 (0.86, 1.30) | 1.36 (1.02, 1.82) |
|  |  | **rs11722146** | **intronic** | **0.29** | **G/A** | **0.31** | **1.06 (0.84, 1.35)** | **1.37 (0.98, 1.91)** |
|  |  | *rs12509517* | *intronic* | *0.29* | *G/C* | *0.31* | *1.07 (0.85, 1.36)* | *1.38 (0.99, 1.93)* |
|  |  | *rs3755867* | *intronic* | *0.32* | *A/G* | *0.57* | *1.08 (0.86, 1.35)* | *1.26 (0.92, 1.72)* |
|  |  | **rs4648090** | **intronic** | **0.14** | **G/A** | **0.98** | **0.64 (0.36, 1.12)** | **1.17 (0.58, 2.34)** |
|  |  | **rs4648110** | **intronic** | **0.20** | **T/A** | **0.99** | **0.65 (0.44, 0.94)** | **1.38 (0.85, 2.23)** |
|  |  | **rs4648127** | **intronic** | **0.06** | **C/T** | **0.84** | **1.07 (0.87, 1.32)*** | **1.17 (0.85, 1.60)*** |
|  |  | *rs4648141* | *intronic* | *0.18* | *G/A* | *0.71* | *0.75 (0.50, 1.13)* | *1.37 (0.80, 2.32)* |

NOTES: Odds ratios (ORs) compared to reference of major allele homozygotes; from logistic regression, minimally adjusted for age, sex, center, and race.

Colon study sample size ranges from: 1926 to 1956, controls; 1527 to 1556, cases.

Rectal study sample size ranges from: 947 to 959, controls; 737 to 754, cases.

Unbolded SNPs failed genotyping, were not in Hardy-Weinberg equilibrium, were monomorphic, or with MAF<0.01 were not carried forward to further analyses.

Italicized SNPs were in high LD (r^2^>0.80) with one or more binned tagging-SNPs and were not carried forward to further analyses.

*Indicates combined analysis of heterozygotes and minor allele homozygotes due to MAF< 0.10.
