# Supplementary material for: Exploring multilocus associations of inflammation genes and colorectal cancer risk using hapConstructor
Source: BMC Med Genet. 2010 Dec 3;11:170. doi: 10.1186/1471-2350-11-170 (PMC3006374; doi:10.1186/1471-2350-11-170)
Supplement: Additional file 2 — Composite genotype logistic regression model associations with colon and rectal cancer identified in hapConstructor. Results of logistic regression models including a multiplicative interaction term for composite genotype associations identified in hapConstructor. [file 1471-2350-11-170-S2.DOC]

Additional file 2. Composite genotype logistic regression model associations with colon and rectal cancer identified in hapConstructor

|  |  | **Colon study** | | | | | | | |
| --- | --- | --- | --- | --- | --- | --- | --- | --- | --- |
|  |  | Controls | |  | Cases | |  |  |  |
|  |  | N | (%) |  | N | (%) | OR* | (95% CI) | |
| IKBKB (rs3747811 T>A) & NFKB1 (rs4648110 T>A) | | | | |  |  |  |  |  |
|  | TT & TT/TA | 504 | 25.8 |  | 430 | 27.6 | 1.00 |  |  |
|  | TT & AA | 23 | 1.2 |  | 17 | 1.1 | 0.85 | (0.45, | 1.62) |
|  | TA/AA & TT/TA | 1369 | 70.0 |  | 1082 | 69.5 | 0.93 | (0.80, | 1.09) |
|  | TA/AA & AA | 60 | 3.1 |  | 27 | 1.7 | 0.54 | (0.34, | 0.87) |
|  | P Interaction |  |  |  | 0.34 |  |  |  |  |
| IL6 (rs2069827 G>T) & NFKB1 (rs4648110 T>A) | | | |  |  |  |  |  |  |
|  | GG & TT/TA | 1544 | 78.9 |  | 1275 | 81.9 | 1.00 |  |  |
|  | GG & AA | 66 | 3.4 |  | 41 | 2.6 | 0.76 | (0.51, | 1.13) |
|  | GT/TT & TT/TA | 329 | 16.8 |  | 237 | 15.2 | 0.89 | (0.74, | 1.06) |
|  | GT/TT & AA | 17 | 0.9 |  | 3 | 0.2 | 0.21 | (0.06, | 0.74) |
|  | P Interaction |  |  |  | 0.06 |  |  |  |  |
| IL6 (rs1800797 G>A) & NFKB1 (rs4648110 T>A) | | | |  |  |  |  |  |  |
|  | GG & TT/TA | 721 | 36.9 |  | 607 | 39.0 | 1.00 |  |  |
|  | GG & AA | 34 | 1.7 |  | 28 | 1.8 | 0.97 | (0.58, | 1.63) |
|  | GA/AA & TT/TA | 1151 | 58.9 |  | 904 | 58.1 | 0.95 | (0.83, | 1.09) |
|  | GA/AA & AA | 49 | 2.5 |  | 16 | 1.0 | 0.40 | (0.23, | 0.72) |
|  | P Interaction |  |  |  | 0.03 |  |  |  |  |
| IKBKB (rs5029748 C>A) & IL6 (rs1800797 G>A) & NFKB1 (rs4648110 T>A) | | | | | | | |  |  |
|  | CC & GG & TT/TA | 386 | 19.7 |  | 334 | 21.5 | 1.00 |  |  |
|  | CC & GG & AA | 21 | 1.1 |  | 18 | 1.2 | 0.97 | (0.51, | 1.86) |
|  | CC & GA/AA & TT/TA | 624 | 31.9 |  | 476 | 30.6 | 0.90 | (0.74, | 1.09) |
|  | CC & GA/AA & AA | 31 | 1.6 |  | 13 | 0.8 | 0.51 | (0.26, | 0.99) |
|  | CA/AA & GG & TT/TA | 335 | 17.1 |  | 272 | 17.5 | 0.93 | (0.75, | 1.15) |
|  | CA/AA & GG & AA | 13 | 0.7 |  | 10 | 0.6 | 0.90 | (0.39, | 2.10) |
|  | CA/AA & GA/AA & TT/TA | 527 | 27.0 |  | 428 | 27.5 | 0.94 | (0.78, | 1.15) |
|  | CA/AA & GA/AA & AA | 18 | 0.9 |  | 3 | 0.2 | 0.20 | (0.06, | 0.67) |
|  | P Interaction |  |  |  | 0.12 |  |  |  |  |
| IL6 (rs1800797 G>A) & NFKB1 (rs4648090 G>A) & NFKB1 (rs4648110 T>A) | | | | | | | |  |  |
|  | GG & GG & TT/TA | 572 | 29.3 |  | 469 | 30.2 | 1.00 |  |  |
|  | GG & GG & AA | 3 | 0.2 |  | 3 | 0.2 | 1.18 | (0.24, | 5.93) |
|  | GG & GA/AA & TT/TA | 149 | 7.6 |  | 138 | 8.9 | 1.13 | (0.87, | 1.47) |
|  | GG & GA/AA & AA | 31 | 1.6 |  | 25 | 1.6 | 0.98 | (0.57, | 1.69) |
|  | GA/AA & GG & TT/TA | 881 | 45.1 |  | 707 | 45.5 | 1.00 | (0.85, | 1.17) |
|  | GA/AA & GG & AA | 4 | 0.2 |  | 2 | 0.1 | 0.64 | (0.12, | 3.53) |
|  | GA/AA & GA/AA & TT/TA | 270 | 13.8 |  | 197 | 12.7 | 0.91 | (0.73, | 1.13) |
|  | GA/AA & GA/AA & AA | 45 | 2.3 |  | 14 | 0.9 | 0.40 | (0.21, | 0.73) |
|  | P Interaction |  |  |  | 0.16 |  |  |  |  |
| IL6 (rs1800797 G>A) & NFKB1 (rs13117745 C>T) & NFKB1 (rs4648110 T>A) | | | | | | | |  |  |
|  | GG & CC & TT/TA | 545 | 27.9 |  | 436 | 28.0 | 1.00 |  |  |
|  | GG & CC & AA | 0 | 0.0 |  | 1 | 0.1 |  |  |  |
|  | GG & CT/TT & TT/TA | 176 | 9.0 |  | 171 | 11.0 | 1.21 | (0.94, | 1.54) |
|  | GG & CT/TT & AA | 34 | 1.7 |  | 27 | 1.7 | 0.99 | (0.59, | 1.67) |
|  | GA/AA & CC & TT/TA | 844 | 43.2 |  | 674 | 43.3 | 1.02 | (0.86, | 1.20) |
|  | GA/AA & CC & AA | 3 | 0.2 |  | 2 | 0.1 | 0.90 | (0.15, | 5.43) |
|  | GA/AA & CT/TT & TT/TA | 307 | 15.7 |  | 230 | 14.8 | 0.95 | (0.77, | 1.18) |
|  | GA/AA & CT/TT & AA | 46 | 2.4 |  | 14 | 0.9 | 0.39 | (0.21, | 0.73) |
|  | P Interaction |  |  |  | 0.06 |  |  |  |  |
|  |  | **Rectal study** | | | | | | | |
|  |  | Controls | |  | Cases | |  |  |  |
|  |  | N | (%) |  | N | (%) | OR | (95% CI) | |
| IKBKB (rs3747811 T>A) & NFKB1 (rs11722146 G>A) | | | | | |  |  |  |  |
|  | TT & GG | 128 | 13.3 |  | 109 | 14.5 | 1.00 |  |  |
|  | TT & GA/AA | 131 | 13.7 |  | 92 | 12.2 | 0.82 | (0.57, | 1.19) |
|  | TA/AA & GG | 357 | 37.2 |  | 228 | 30.2 | 0.77 | (0.56, | 1.05) |
|  | TA/AA & GA/AA | 343 | 35.8 |  | 325 | 43.1 | 1.11 | (0.83, | 1.50) |
|  | P Interaction |  |  |  | 0.01 |  |  |  |  |
| IL6 (rs1800797 G>A) & NFKB1 (rs11722146 G>A) | | | |  |  |  |  |  |  |
|  | GG & GG | 203 | 21.2 |  | 146 | 19.4 | 1.00 |  |  |
|  | GG & GA/AA | 216 | 22.5 |  | 171 | 22.7 | 1.09 | (0.81, | 1.46) |
|  | GA/AA & GG | 282 | 29.4 |  | 191 | 25.3 | 1.01 | (0.75, | 1.34) |
|  | GA/AA & GA/AA | 258 | 26.9 |  | 246 | 32.6 | 1.38 | (1.04, | 1.83) |
|  | P Interaction |  |  |  | 0.25 |  |  |  |  |
| IL6 (rs1800797 G>A) & NFKB1 (rs3821958 A>G) | | | |  |  |  |  |  |  |
|  | GG & AA | 129 | 13.5 |  | 108 | 14.3 | 1.00 |  |  |
|  | GG & AG/GG | 290 | 30.2 |  | 209 | 27.7 | 0.86 | (0.63, | 1.18) |
|  | GA/AA & AA | 199 | 20.8 |  | 125 | 16.6 | 0.81 | (0.57, | 1.14) |
|  | GA/AA & AG/GG | 341 | 35.6 |  | 312 | 41.4 | 1.15 | (0.85, | 1.56) |
|  | P Interaction |  |  |  | 0.02 |  |  |  |  |
| *Logistic regression models adjusted for age, center, race, and sex. | | | | | | |  |  |  |
